# A Comparison of Amplitude-and Time-Dependent Cyclic Deformation Behavior for Fully-Austenite Stainless Steel 316L and Duplex Stainless Steel 2205

**DOI:** 10.3390/ma14195594

**Published:** 2021-09-26

**Authors:** Shaohua Li, Wenchun Jiang, Xuefang Xie, Zhilong Dong

**Affiliations:** State Key Laboratory of Heavy Oil Processing, College of New Energy, China University of Petroleum (East China), Qingdao 266580, China; joshuali_upc@126.com (S.L.); xiexuefang_upc@126.com (X.X.); dongzhilong0318@126.com (Z.D.)

**Keywords:** amplitude-dependent, time-dependent, cyclic deformation behavior, duplex-phase microstructure, nitrogen

## Abstract

Austenite and duplex stainless steels are widely used in engineering, and the latter exhibits a more excellent combination of mechanical properties and corrosion resistance due to the coexistence of austenite and ferrite and higher nitrogen. However, fatigue failure still threatens their structural integrity. A comprehensive comparison of their cyclic deformation behavior is a major foundation to understand the role of duplex-phase microstructure and nitrogen in the safety assessment of engineering components. Thus, in this paper, the cyclic deformation behavior of fully-austenitic stainless steel 316L and duplex stainless steel 2205 was studied by a series of low cycle fatigue tests with various strain amplitudes, loading rates and tensile holding. A theoretical mechanism diagram of the interaction between nitrogen and dislocation movements during cyclic loads was proposed. Results show that the cyclic stress response of 2205 was the primary cyclic hardening, followed by a long-term cyclic softening regardless of strain amplitudes and rates, while an additional secondary hardening was observed for 316L at greater strain amplitudes. Cyclic softening of 2205 was restrained under slower strain rates or tensile holding due to the interaction between nitrogen and dislocations. The cyclic plasticity of 2205 started within the austenite, and gradually translated into the ferrite with the elevation of the cyclic amplitude, which lead to a decreased hardening ratio with the increase in amplitude and a shorter fatigue life for a given smaller plastic strain amplitude.

## 1. Introduction

Duplex stainless steel (DSS), containing nearly equal volume fractions of austenite and ferrite, combines the advancements of single-phase stainless steels, such as their excellent mechanical proprieties, corrosion resistance, weldability, etc. Thus, it has been increasingly employed as the structural material in many industries, including the petrochemical fields, oceaneering and nuclear power engineering [1]. However, fatigue failure due to frequent start-up and shut-down operations, or the cyclic loads in service, still threatens the structural integrity of these engineering components [2,3]. Compared with single-phase stainless steel, the local plastic activities of each constitutive phase and their interaction lead to a more complex fatigue performance. In addition, the nitrogen content for DSS is usually much higher than that of conventional single-phase stainless steel to improve the phase ratio and mechanical properties of its weld joints [4,5,6], for example, it is up to 0.3 wt.% for DSS 2507, but only 0.04 wt.% for the austenitic stainless steel (ASS) 316L. The forceful interaction between the nitrogen, as the interstitial solute atom, and the dislocation movement has a significant effect on the cyclic plasticity deformation. Hence, a comprehensive comparison of cyclic deformation behavior for single-phase stainless steel and DSS is a major foundation to understand the role of duplex-phase microstructure and nitrogen in the safety assessment of engineering components.

During the past several decades, considerable efforts have been focused on the cyclic deformation behavior and corresponding microscopic physical mechanism of ASS, including types of Cr-Ni-Fe alloy (such as 316L [7,8,9,10,11,12,13,14,15], 304 [16,17,18], Sancro 25 [19,20,21,22,23] and Inconel 617 [24]) and rich-Mn alloy (Hadfield steel [25,26,27]). It has been widely recognized that transient cyclic hardening followed by long-term cyclic softening is the main macroscopic characteristic for ASSs under strain-controlled cyclic loading at the room temperature. Particularly for ASSs with relatively higher-content alloy elements, such as the 316LN and Sancro 25, continuous cyclic softening without any hardening was observed at lower cyclic amplitudes [15,21,22,23], but under larger cyclic amplitudes, the materials may exhibit additional secondary cyclic hardening behavior [12,13,16,18]. Meanwhile, the evolution of dislocation configurations during the cyclic process of ASS has been continuously explored. The dislocation multiplication or tangles by multiple slip usually increase the resistance to plastic deformation due to the enhanced short-range interaction between dislocations, which leads to the macroscopic primary cyclic hardening behavior [9,10,11]. Then, the dislocation annihilation or dynamic recovery form lower-energetic configurations, such as dislocation walls/channels or cells, leading easily to the localization of plastic deformation and further resulting in the cyclic softening behavior [9,10,11]. The deformation-induced martensitic transformation is regarded as the main mechanism for the secondary cyclic hardening of ASS under larger cyclic amplitudes [16]. In addition, at elevated temperatures, the dynamic strain aging effect, creep, oxidation and their interaction play a more important role in cyclic deformation behavior of the ASS, and lead to more pronounced cyclic hardening behavior and a shorter fatigue life [19,20,21,22,23,24].

The cyclic deformation behavior and mechanism of the DSS are much different to that of the single-phase stainless steel due to the effect of the duplex-phase microstructure and the high-content nitrogen. The strain-controlled uniaxial low cycle fatigue (LCF) tests for the hot-rolled or solution-treated DSSs were carried out in previous research [28,29,30,31,32,33], which found that the macroscopic cyclic deformation behavior of DSS can be characterized by primary cyclic hardening at first few cycles, followed by long-term cyclic softening, regardless of the strain amplitudes. Li et al. [34] further investigated the cyclic deformation behavior of the DSS S32205 under torsional fatigue loadings, and reported that the primary hardening and following softening were still observed when the strain amplitude was greater than 0.3%, but a continuous softening up to final rupture was exhibited for lower strain amplitudes. In addition, the dislocation patterns of each constitutive phase during cyclic loading were examined by transmission electron microscope (TEM) observation. A significant transition of the deformation-bearing phase from austenite to ferrite with the elevation of cyclic amplitude has been found [30,31,32,33,34]. Mateo et al. [30] divided the cyclic mechanical response of the AISI329 under different amplitudes into three regimes, i.e., austenitic-like behavior for the lower amplitudes, mixed austenitic-ferritic behavior for medium amplitudes and ferritic-like behavior for larger amplitudes. Li et al. [34] pointed out that the formation of low-energy dislocation configurations within the ferrites, including the dislocation wall/channel and cell, dominates the macroscopic cyclic softening behavior, while most of the austenitic grains still maintain the planar slip mode even under greater cyclic amplitudes. However, although the TEM observation gave much information about the dislocation patterns of individual phase, the grains-level local deformation distribution, which reflects the interaction between two phases, still cannot be identified. Further, the previous researches were limited to the cyclic performance of the DSS at continuous cyclic loading with a constant rate, and the practical loading of engineering components is usually varied-rate with holding during normal serving conditions. Thus, the time-dependent cyclic properties of the DSS still require extensive investigation for a better safety assessment. Furthermore, a direct and comprehensive comparison of the cyclic deformation behavior between the DSS and ASS is also required to deeply understand the role of duplex-phase structure and nitrogen in cyclic deformation behavior and fatigue life of the material.

Therefore, in this study, a series of LCF tests with various strain amplitudes, loading rates and tensile holding for both the ASS 316L and DSS 2205 were firstly carried out to comprehensively understand their cyclic deformation behavior. The distribution of microscopic plastic behavior within two phases of 2205 under different amplitudes was observed by electron back scatter diffraction (EBSD) analysis, and the correlations with cyclic mechanical behavior and fatigue life were revealed. Finally, a theoretical mechanical model of the interaction of nitrogen and dislocation movements during cyclic loading was proposed to explore the time dependence of cyclic mechanical responses.

## 2. Materials and Methods

### 2.1. Material Description

**Figure 1:**
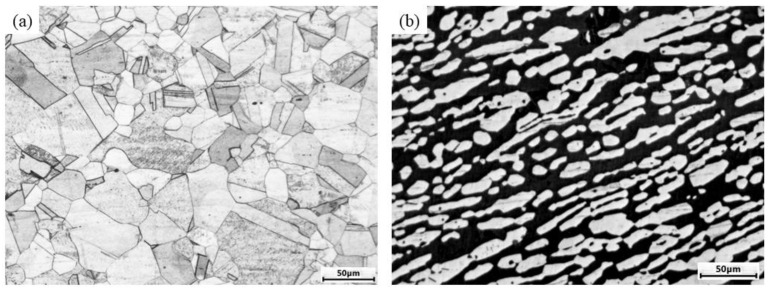
OM-based microstructures of (**a**) 316L and (**b**) 2205.

The ASS 316L and DSS 2205 were evaluated simultaneously in this study, and their chemical compositions are listed in Table 1. It can be seen that the nitrogen content of 2205 is much higher than that of 316L. The heat treatment for two materials before the mechanical tests were identical, i.e., the solution was treated at 1070 °C for 2 h and followed by water cooling. The optical microscope (OM) was used to observe microstructures after the etch of 1.5 g K_2_S_2_O_5_ + 15 mL HCL + 85 mL H_2_O for 2205 and aqua regia for 316L, as shown in Figure 1. Only the austenitic grains with some annealing twins were observed for 316L, while both the island-like austenitic phase and ferritic matrix phase with a ratio of near 48/52 were identified for 2205, and for two solution-treated materials no significant precipitation was found. The average diameter of grains was 7.52 μm for 316L, and about 7.39 μm and 8.03 μm for austenite and ferrite in 2205, respectively. 

### 2.2. LCF Mechanical Tests

**Figure 2:**
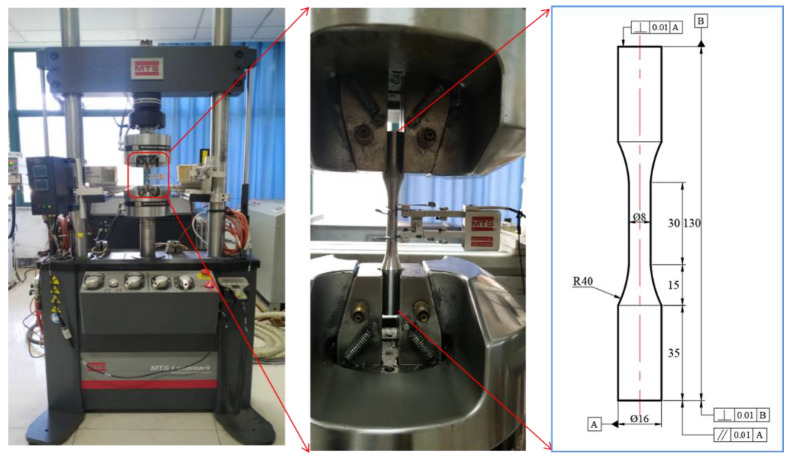
Experimental setup of LCF tests and detailed dimensions of specimens.

The strain-controlled LCF tests for the cylindrical hourglass polished samples with a parallel length of 30 mm and a gauge diameter of 8 mm were conducted on a MTS servo-hydraulic fatigue machine (MTS Inc., Huntsville, AL, USA). The deformation in the parallel section was monitored and controlled by an axial extensometer with a clip gauge of 20 mm, and the magnitudes of axial loads imposed on the specimen were measured by a force sensor. The experimental setup and detailed dimensions of specimens are shown in Figure 2. 

**Figure 3:**
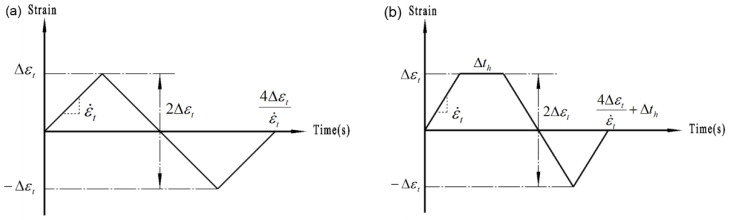
Schematic loading process: (**a**) continuous LCF and (**b**) LCF with tensile holding.

Two types of tests were designed, i.e., the continuous LCF (Figure 3a), and the LCF with tensile holding (Figure 3b). The holding time was considered as 30 s. The continuous LCF tests were further designed into two groups, i.e., the tests with a constant strain rate ${\dot{\varepsilon }}_{t}$ of 5 × 10^−3^s^−1^ but different strain amplitudes $\Delta \varepsilon_{t}$ varied from ±0.3% to ±1.0%, and the tests with a constant $\Delta \varepsilon_{t}$ of ±0.6% and different ${\dot{\varepsilon }}_{t}$ varied from 5 × 10^−4^ s^−1^ to 1 × 10^−2^ s^−1^. In addition, the repeated tests were conducted randomly to ensure the reliability of experimental results. A total of 26 LCF tests are listed in Table 2. 

### 2.3. Microstructure Observation

To reveal the cyclic deformation response within two constitutive phases, EBSD analysis was conducted on the cross-sectional sample cut by the line cutting technique. The samples were electric etched by using a 40 wt% sodium hydroxide solution with a direct-current voltage of 3 V for 10 s until the tested surface became straw yellow. The observed plane was perpendicular to the loading direction. In order to figure out the local plastic deformation of constitutive phases under different cyclic amplitudes, the analyzed samples were cut from the fatigue specimens after 1000 cycles under the strain amplitudes of 0.3% and 1.0%. To maximize the amount of backscatter electrons and increase the probability of diffraction events, the sample was mounted on the scanning electron microscope chamber with the sample tilted at 70° to the phosphor detector. An accelerating voltage of 20 kV and a current of 2.4 nA were applied in the EBSD measurement. A step size of 0.5 μm was taken to assess the local misorientation distribution. HKL Channel 5 was then applied to process the EBSD data.

## 3. Results 

### 3.1. Amplitude-Dependent Cyclic Stress Responses

**Figure 4:**
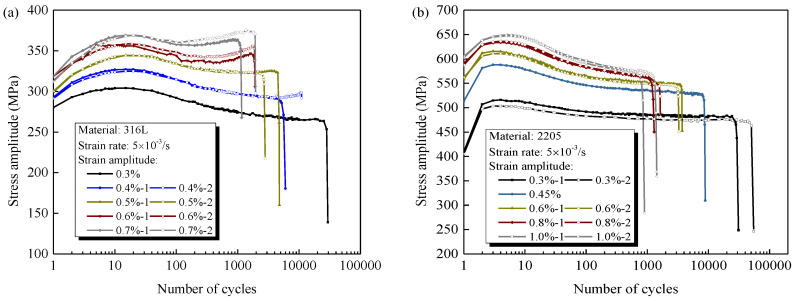
Cyclic stress amplitude for (**a**) 316L and (**b**) 2205 under different strain amplitudes with a constant strain rate of 5 × 10^−3^ s^−1^.

Figure 4a,b depicts the evolution of cyclic stress amplitude for 316L and 2205 under various strain amplitudes with a constant rate 5 × 10^−3^ s^−1^, respectively. The cyclic stress responses from the repeated experiments are relatively identical, implying that the experimental results are sufficiently trustworthy. It is obvious that the cyclic hardening/softening behavior of 316L depends significantly on the strain amplitude, i.e., the primary cyclic hardening following by moderate softening up to final rupture for lower strain amplitudes (<0.6%), but a secondary hardening before fatigue rupture is clearly identified for greater strain amplitudes. However, for 2205, the primary cyclic hardening and subsequent softening are applied to all the investigated strain amplitudes, and no additional hardening is observed even when the amplitude is up to ±1.0%. These findings are consistent with the experimental results obtained in the previous researches [28,29,30,31,32,33].

**Figure 5:**
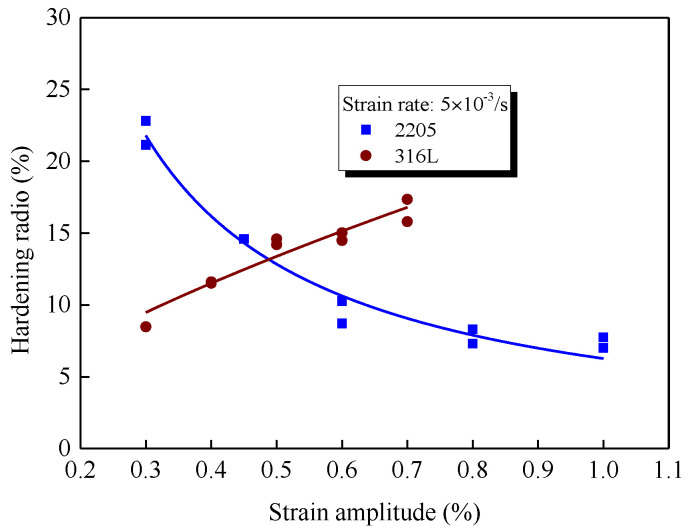
Hardening ratios under different strain amplitudes for 2205 and 316L.

To better understand the cyclic hardening behavior, a hardening ratio is defined as the ratio of maximum stress amplitude and the stress amplitude at the first cycle, and its evolution with strain amplitudes for two materials is shown in Figure 5. For 316L, the hardening ratio increased with the elevation of strain amplitude. As introduced before, the macroscopic cyclic hardening behavior is mainly due to the multiplication of the dislocation caused by the plastic deformation, which promotes critical slip resistance by enhancing the short-range interaction between dislocations [9,10,11]. Thus, for 316L, it is easily accepted that more plastic deformation within one cycle for higher amplitudes indicates more significant cyclic hardening behavior. It is very interesting that for 2205, the hardening ratio is decreased with the increase in amplitude. Besides, it is also noted that the hardening rate of 2205 is also much faster than that of 316L under the same strain amplitude. The unusual cyclic hardening behavior of the 2205 is closely related to the microscopic local deformation within two constitutive phases, which will be discussed in Section 4.1.

### 3.2. Rate-Dependent Cyclic Stress Response

**Figure 6:**
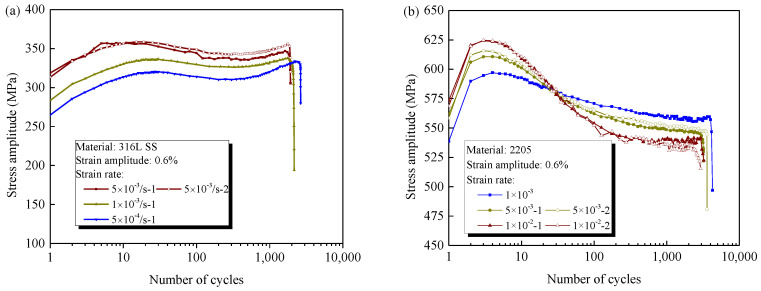
Cyclic stress amplitude for (**a**) 316L and (**b**) 2205 under different strain rates with a constant strain amplitude.

The rate-dependent cyclic deformation behavior of materials is illustrated by the evolution of stress amplitude under different strain rates, as shown in Figure 6a,b. Obviously, the stress responses of both materials vary with the strain rate, but there is a significant difference that must be addressed, i.e., the stress amplitudes of 316L increase with the elevation of strain rate, and its evolution during the whole fatigue life at different rates is nearly parallel. Based on kinetic mechanics theory, for a given total strain, more elastic deformation is required for a material under a faster loading rate due the hysteresis of plastic deformation, resulting in a greater stress response. The nearly parallel evolution of the stress response for 316L under different strain rates implies that the strain rate has little effect on the evolution of the microstructures under cyclic loading. However, for 2205, an asynchronous effect of strain rate on the cyclic hardening and softening behavior is observed, i.e., the stresses at the cyclic hardening stage are enhanced with the increase in strain rate, but the saturated cyclic stresses become lower due to more pronounced cyclic softening. 

### 3.3. Cyclic Stress Response under the Effect of Holding 

**Figure 7:**
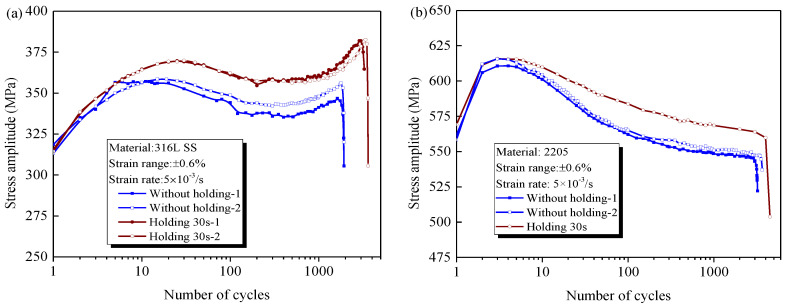
Cyclic stress amplitude for (**a**) 316L and (**b**) 2205 under effect of holding at strain rate of 5 × 10^−3^ s^−1^.

Figure 7a,b describe the evolution of stress amplitude for 316L and 2205 under the effect of tensile holding for 30 s, respectively. For 316L, the cyclic hardening behavior is enhanced by the tensile holding, leading to a greater stress amplitude at the beginning of the cyclic softening, but its subsequent evolution is still nearly parallel with that of the continuous LCF tests, including the softening and secondary cyclic hardening stages. This implies that tensile holding has little effect on the cyclic softening and secondary hardening behaviors of 316L. However, it is completely different for 2205. The effect of tensile holding on the cyclic hardening is nearly negligible, whereas cyclic softening behavior is inhibited significantly by the tensile holding. 

**Figure 8:**
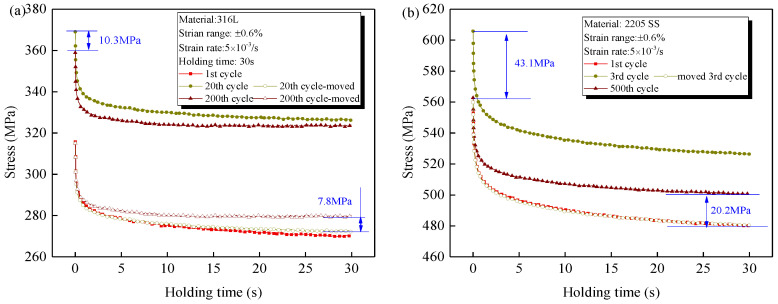
Evolution of stress relaxation behaviour for (**a**) 316L and (**b**) 2205.

The stress response is determined by the magnitude of elastic strain. For the tensile-holding strain-controlled LCF, the holding provides enough time for the dislocation slip movements. Thus, the hysteretic plastic deformation forms gradually and some elastic strain is recovered when the total strain is constant, resulting in stress relaxation behavior. This is to say, the stress relaxation reflects the magnitude of viscous stress. Figure 8a,b compare the stress relaxation behavior at different cycles for 316L and 2205, respectively. For the convenient comparison, the stress relaxation curves at different cycles are moved to a common starting point, as shown by the hollow dots. For both materials during the cyclic hardening, although the stress amplitude increases significantly due to the cyclic hardening, the stress relaxation behavior is still not changed, which implies that the increase in dislocation density has little effect on the viscous properties of the material. However, a decreased stress relaxation behavior is clearly observed for two materials under the cyclic softening process, for example, the relaxed stresses of 316L at the 200th cycle is 7.8 MPa lower than that at the 2nd cycle, when the stress amplitude is softened by 10.3 MPa. As for 2205, the relaxation behavior at the 500th cycle is also decreased significantly comparing with that at the 1st cycle, although their stress amplitudes are all 562 MPa. This indicates the decrease in viscous stress is an important factor for the softening of stress amplitude.

It has been widely recognized that for 316L, the dislocation density is increased significantly during cyclic hardening, but the patterns of most dislocations still remain planar. At the following cyclic softening, a dynamic recovery of dislocation to form lower-energetic configurations is observed, such as the dislocation walls/channels or cells [9,10,11]. Combining the evolution of the stress relaxation behavior in Figure 8a, it is concluded that the viscous stress depends more on the dislocation morphology than the dislocation density. In addition, it is also worth noting that the decreased viscous stress is much lower than the softened stress amplitude for 2205, but it is the greatest for 316L, which indicates that besides the viscous softening induced by the dynamic recovery of dislocations, there must be another mechanism for the cyclic softening of 2205, which will be discussed in Section 4.2.

## 4. Discussion

### 4.1. Effect of Duplex-Phase Structure on Cyclic Deformation

**Figure 9:**
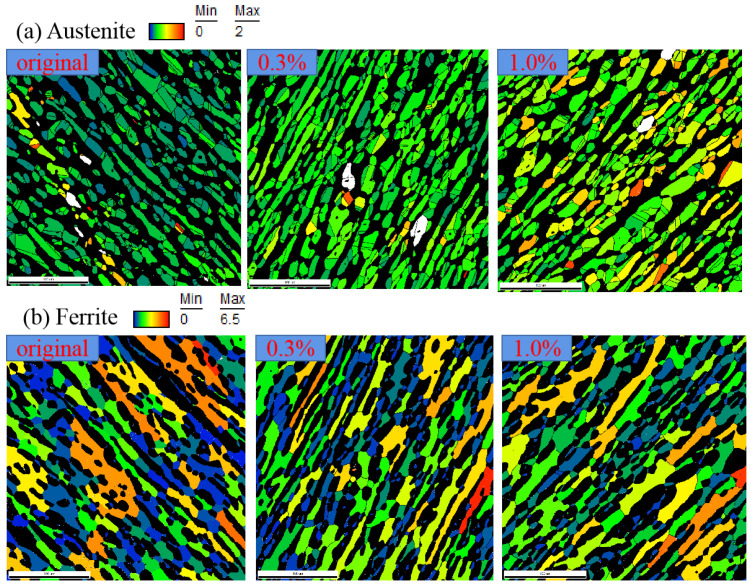
Distribution of GAM within two constitutive phases for 2205 under different strain amplitudes: (**a**) austenite and (**b**) ferrite.

**Figure 10:**
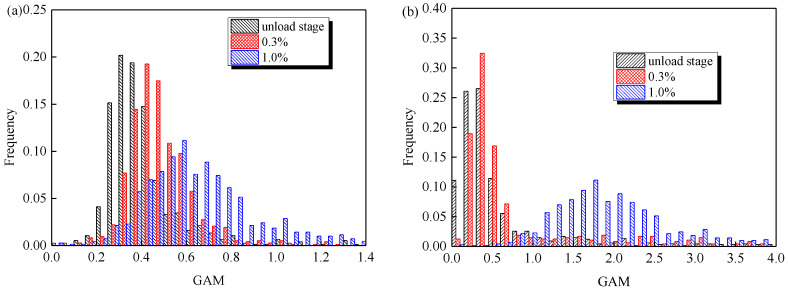
Statistics of GAM distribution within constituent phases after different strain amplitudes: (**a**) austenite and (**b**) ferrite.

To illustrate the effect of duplex phases on cyclic deformation behavior, the microscopic plastic deformation within two phases of 2205 after different strain amplitudes was firstly observed by EBSD. Figure 9 shows the distribution of grain average misorientation (GAM), defined as the average misorientation between all neighboring data points in the grain. It was clearly found that for austenite, the magnitudes of GAM increased with the elevation of applied amplitude, even under the smaller amplitude of 0.3%. However, the magnitude of GAM for ferrite under the strain amplitude of 0.3% was very slightly greater than that at the unloading stage, while the evolution became pronounced for the strain amplitude of 1.0%. This evolution of GAM with strain amplitude is presented more significantly by the statistical results, and the average GAMs of austenite under three amplitudes were 0.32°, 0.45° and 0.63°, but they were 0.35°, 0.37° and 1.8° for ferrite, as shown in Figure 10. As a result, it is concluded that the cyclic plasticity of the DSS starts within the austenite, and gradually translates into the ferrite with the elevation of the applied cyclic amplitude. 

**Figure 11:**
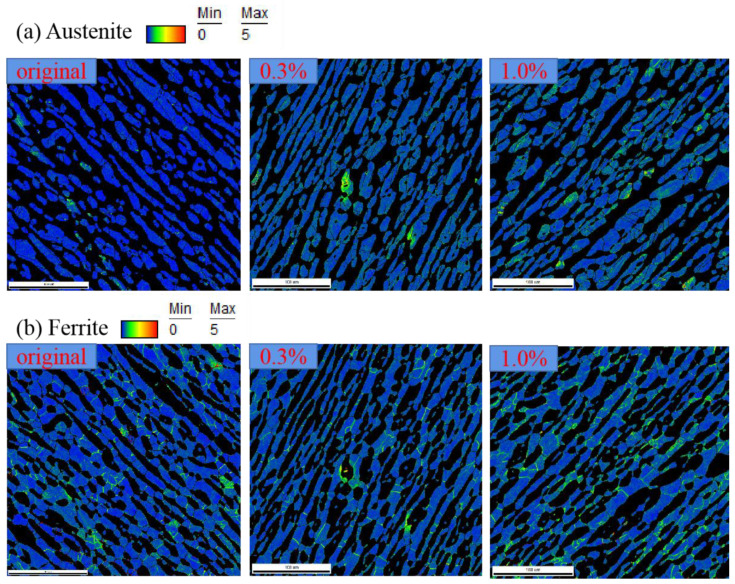
Distribution of KAM within two constitutive phases for 2205 under different strain amplitudes: (**a**) austenite and (**b**) ferrite.

The intra-grain level plastic deformation within two phases of 2205 is illustrated by the distribution of Kernel average misorientation (KAM) which is defined as the average misorientation between the data point and all of its neighbors, as shown in Figure 11. The higher KAM indicates the greater local microscopic plastic deformation. Obviously, the plastic deformation within the austenite was preferentially formed at the phase boundaries, while it mainly concentrated at the ferritic grain boundaries for the ferrite. The deformation incompatibility resulting from the crystal misorientation or phases properties is the main reason for the concentration of plastic deformation at phase boundaries or grain boundaries. As the austenite is the relatively softer phase, at the phase boundaries the plastic deformation preferentially occurred at the sides of austenitic grains.

**Figure 12:**
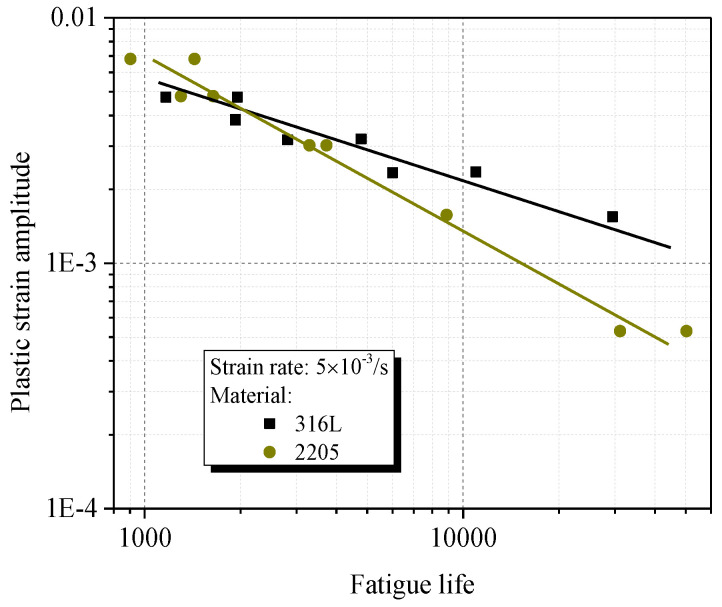
Relationship between fatigue life and plastic strain amplitudes.

Both the distribution of GAM and KAM within two phases of 2205 indicate that the cyclic plasticity of 2205 starts within the austenite, and gradually translates into the ferrite with the elevation of cyclic amplitude. Due to the differences in lattice structure, the hardening property of austenite (face-centered cubic) is greater than that of ferrite (body-centered cubic), which has been proved by the cyclic nanoindentation tests in Ref [35]. Thus, the hardening ratio was decreased with the elevation of strain amplitude for 2205 due to the change in deformation-bearing phases, as shown in Figure 5. Besides, the loading sharing behavior between two constitutive phases also lead to the heterogeneous distribution of plastic deformation, particularly under smaller cyclic amplitudes. Thus, for a given smaller plastic strain amplitude, the local plastic strain of the austenite for 2205 is much greater than that in 316L, in which the plastic deformation is relatively homogeneous. As a result, the fatigue life of 2205 is much shorter than that of 316L, as shown in Figure 12.

### 4.2. Effect of Nitrogen on Cyclic Deformation

**Figure 13:**
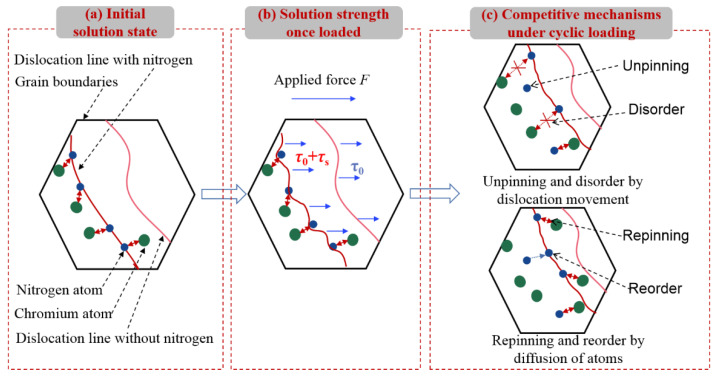
Evolution of interaction between nitrogen atoms and dislocation movements under cyclic loading.

The effect of nitrogen on the cyclic deformation behavior is mainly presented by its short-range interaction with the dislocation movements, as sketchily shown in Figure 13. For the solution-treated material, the high temperature makes it easy for nitrogen to diffuse towards the dislocations where lots of defects exist, as shown in Figure 13a. Once a large enough load *F* is applied to the grain, the plastic deformation occurs by the slip movements of dislocations. For the dislocations without nitrogen, the critical slip resistance *τ*_0_ mainly includes the intrinsic lattice friction stress (Peirls-Nabarro stress) and the stresses by the short-range interaction between nearby dislocations and grain boundaries [36,37,38]. However, for the dislocation with nitrogen, an additional stress *τ*_s_ is required to overcome the hindrance of nitrogen induced by both the pinning of the Cottrell atmosphere and Cr-N short-range order (SRO) [39,40], as shown in Figure 13b. Then, upon further cyclic loading, two kinds of competitive mechanisms jointly determine the mechanical response, as shown in Figure 13c. The first one is the unpinning of dislocations from the nitrogen atoms when they pass through the nitrogen, or the disorder of Cr-N SRO with the movement of nitrogen. This reduces the critical slip resistance for the subsequent dislocations movements. Another one is the repining induced by the diffusion of nitrogen, or the reordering of the SRO with surrounding chromium atoms, which increases critical slip resistance. Therefore, for 2205, apart from the viscous softening, additional cyclic softening is observed due to the unpinning and disorder of nitrogen, as shown in Figure 8b. On the other hand, the lower loading rate or the tensile holding provides more time for the repinning and reordering of nitrogen, which leads to decreased cyclic softening behavior, as shown in Figure 6b and Figure 7b.

**Figure 14:**
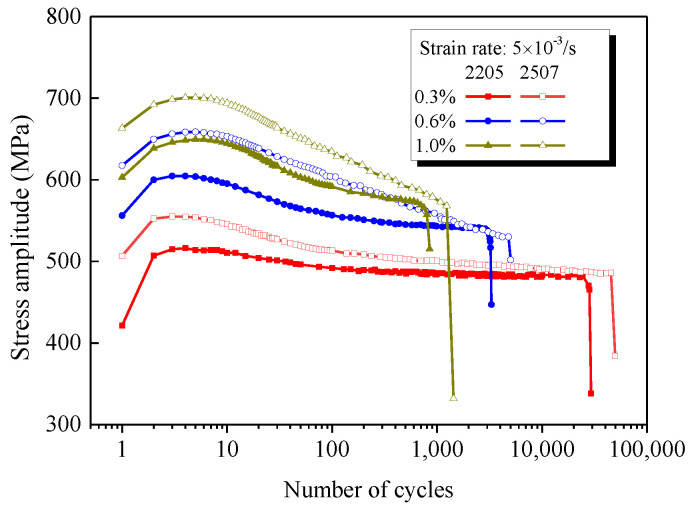
Comparison of cyclic stress responses of 2205 and 2507 under different strain amplitudes with a constant rate 5 × 10^−3^ s^−1^.

It is very difficult to perform an in-situ observation of the interaction between nitrogen atoms and dislocation movement under cyclic loading, particularly for the polycrystalline material. To prove the accuracy of the mechanism model shown in Figure 13, the cyclic mechanical responses of DSS 2507, which has a higher nitrogen content (0.3wt.%) than that of 2205 (0.15wt.%), were investigated by the LCF tests. Note that the dimension of specimens and experimental setup are consistent with those of 2205, as introduced in Section 2.2. The comparison of cyclic stress responses of 2205 and 2507 is shown in Figure 14. Obviously, the level of stress amplitude of 2507 is much greater than that of 2205 for a given strain amplitude, but the cyclic softening is more pronounced for all investigated strain amplitudes. This is consistent with the proposed theoretical mechanism model, i.e., the addition of nitrogen increases the strength of the material due to more pinning and Cr-N SRO; at the same time, the unpinning and disorder are also increased during cyclic loading, leading to a more pronounced cyclic softening.

## 5. Conclusions

In this paper, the time- and amplitude-dependent cyclic deformation behavior for both the DSS 2205 and ASS 316L were investigated by a series of LCF tests. The distribution of the microscopic plastic deformation of two phases for 2205 under various cyclic amplitudes was observed by EBSD, and a diagram of the theoretical mechanism of interaction between nitrogen and dislocation movements during cyclic loading was proposed. In the future, the initiation mechanism of fatigue micro-cracks for 2205 due to the effects of duplex-phase microstructure and nitrogen is worth more attention. The main conclusions are listed as follows:

(1) The cyclic deformation behavior of 2205 was exhibited as the primary cyclic hardening followed by long-term cyclic softening, regardless of strain amplitudes and rates, while an additional secondary hardening was observed for 316L at greater strain amplitudes.
(2) Decreased stress relaxation during cyclic loading was observed for both 316L and 2205. However, it was more significant for the 2205 due to the unpinning of dislocations from the nitrogen and the disorder of Cr-N SRO.
(3) The cyclic softening of 2205 was inhibited by slower loading rates and holding, due to the repining of dislocations by the nitrogen and the reorder of nitrogen with surrounding chromium.
(4) The cyclic plasticity of 2205 started within the austenite, and gradually translated into the ferrite with the elevation of the applied cyclic amplitude. This lead to the severely heterogeneous distribution of local plastic deformation among the two phases, particularly for smaller amplitudes, which further resulted in a faster hardening rate and shorter fatigue life compared with 316L.

## Figures and Tables

**Table 1:** Chemical compositions of 316L and 2205 SS (wt.%).

Element	C	Cr	Ni	Mn	S	P	Si	Mo	N	Fe
316L	0.025	16.2	10.2	1.4	0.025	0.025	0.4	2.1	0.043	Rest
2205	0.02	21.8	5.7	1.4	0.002	0.023	0.4	-	0.15	Rest

**Table 2:** Experimental details for all LCF tests.

ID	Material	Amplitude	Rate (s^−1^)	Holding Time (s)	Number of Specimens
1~3	316L/2205	0.3%	5 × 10^−3^	0	1(316L)/2(2205)
4,5	316L	0.4%	5 × 10^−3^	0	2
6	2205	0.45%	5 × 10^−3^	0	1
7,8	316L	0.5%	5 × 10^−3^	0	2
9~12	316L/2205	0.6%	5 × 10^−3^	0	2/2
13,14	316L	0.7%	5 × 10^−3^	0	2
15,16	2205	0.8%	5 × 10^−3^	0	2
17,18	2205	1.0%	5 × 10^−3^	0	2
19	316L	0.6%	5 × 10^−4^	0	1
20,21	316L/2205	0.6%	1 × 10^−3^	0	1/1
22,23	2205	0.6%	1 × 10^−2^	0	2
24~26	316L/2205	0.6%	5 × 10^−3^	30	2(316L)/1(2205)

## Data Availability

Data sharing not applicable.

## Abbreviations

DSS (duplex stainless steel); ASS (austenitic stainless steel); LCF (low cycle fatigue); TEM (transmission electron microscope); EBSD (electron back scattered diffraction); OM (optical microscope); GAM (grain average misorientation); KAM (Kernel average misorientation); SRO (short-range order).
